# Identification of the potential regulatory interactions in rheumatoid arthritis through a comprehensive analysis of lncRNA-related ceRNA networks

**DOI:** 10.1186/s12891-023-06936-3

**Published:** 2023-10-09

**Authors:** Mingyi Yang, Yani Su, Haishi Zheng, Ke Xu, Qiling Yuan, Yongsong Cai, Yirixiati Aihaiti, Peng Xu

**Affiliations:** Department of Joint Surgery, HongHui Hospital, Xi’an Jiaotong University, Xi’an, Shaanxi 710054 China

**Keywords:** Rheumatoid arthritis, Synovial, lncRNA, ceRNA, Enrichment

## Abstract

**Objective:**

This study aimed at constructing a network of competing endogenous RNA (ceRNA) in the synovial tissues of rheumatoid arthritis (RA). It seeks to discern potential biomarkers and explore the long non-coding RNA (lncRNA)-microRNA (miRNA)-messenger RNA (mRNA) axes that are intricately linked to the pathophysiological mechanisms underpinning RA, and providing a scientific basis for the pathogenesis and treatment of RA.

**Methods:**

Microarray data pertaining to RA synovial tissue, GSE103578, GSE128813, and GSE83147, were acquired from the Gene Expression Omnibus (GEO) database (http://www.ncbi.nlm.nih.gov/geo). Conducted to discern both differentially expressed lncRNAs (DELncRNAs) and differentially expressed genes (DEGs). A ceRNA network was obtained through key lncRNAs, key miRNAs, and key genes. Further investigations involved co-expression analyses to uncover the lncRNA-miRNA-mRNA axes contributing to the pathogenesis of RA. To delineate the immune-relevant facets of this axis, we conducted an assessment of key genes, emphasizing those with the most substantial immunological correlations, employing the GeneCards database. Finally, gene set enrichment analysis (GSEA) was executed on the identified key lncRNAs to elucidate their functional implications in RA.

**Results:**

The 2 key lncRNAs, 7 key miRNAs and 6 key genes related to the pathogenesis of RA were obtained, as well as 2 key lncRNA-miRNA-mRNA axes (KRTAP5-AS1-hsa-miR-30b-5p-PNN, XIST-hsa-miR-511-3p/hsa-miR-1277-5p-F2RL1). GSEA of two key lncRNAs obtained biological processes and signaling pathways related to RA synovial lesions.

**Conclusion:**

The findings of this investigation hold promise in furnishing a foundational framework and guiding future research endeavors aimed at comprehending the etiology and therapeutic interventions for RA.

**Supplementary Information:**

The online version contains supplementary material available at 10.1186/s12891-023-06936-3.

## Introduction

Rheumatoid arthritis (RA) constitutes a systemic inflammatory ailment typified by manifestations such as peripheral joint pain, swelling, and stiffness. Its pathophysiological trajectory prominently features synovial inflammation. Indeed, RA ranks as the most prevalent category of inflammatory arthritis. This disease exerts its impact upon the synovial cells and chondrocytes within the articulating joints, thereby precipitating inflammation of the synovial lining, damage to cartilaginous structures, and erosion of bone tissues. The distinctive hallmark of RA is the emergence of locally infiltrative synovial tissue, a phenomenon intimately associated with the progression of the ailment [1]. Clinically, RA manifests a spectrum of severity, with consequences ranging from mild, self-limited arthritic symptoms to rapidly evolving, multi-systemic inflammation [2]. RA may manifest across all age groups, yet its incidence predominantly peaks between the ages of 40–70 years, displaying a male-to-female ratio of approximately 2.5:1. Furthermore, its prevalence escalates with advancing age, exhibiting a global average prevalence rate of approximately 0.5-1.0% [2]. While the etiology of RA remains incompletely elucidated, genetic factors contribute substantially, accounting for up to 60% of disease susceptibility, with sex and smoking being additional noteworthy risk determinants. RA carries the potential for irreversible joint impairment and physical debilitation, and it exerts a profound societal burden. Regrettably, no precise or radically curative interventions exist. Latest research in RA therapy has found that targeted exosome therapy imparts cell-free DNA (cfDNA) clearance and macrophage polarization to improve RA [3]. The FGF pathway is a critical signaling pathway in relapse RA. Targeted tissue-specific inhibition of FGF10/FGFR1 may provide new opportunities to treat patients with relapse RA [4].

Microarray technology yields a wealth of biomarkers and predictive data, thereby affording invaluable research targets for the diagnosis and treatment of diseases [5].The incorporation of microarray technology into gene expression research has engendered a novel avenue for investigating the mechanisms, diagnoses, treatments, and prognoses of complex diseases. By facilitating genome-wide investigations, microarray technology plays an instrumental role in unraveling the underlying mechanisms governing numerous human maladies through the functional annotation of the genome [6]. Over the past few decades, bioinformatics has risen to prominence, emerging as an integral component of life sciences development and pioneering the forefront of life science research. Its significance within the domain of medical research has witnessed a steady ascent. Recent years have witnessed the widespread application of microarray technology and bioinformatics analysis for the identification of potential disease biomarkers, thereby laying the groundwork for the exploration of disease molecular mechanisms [7]. In contemporary times, microarray technology in conjunction with bioinformatics analysis has successfully pinpointed potential biomarkers for a myriad of diseases, including but not limited to colorectal cancer, varicocele, systemic sclerosis, oral squamous cell carcinoma, and RA [7–11].

Numerous genes associated with RA have been identified as potential biomarkers in the pathogenesis of the disease, utilizing bioinformatics techniques. Nonetheless, scant attention has been devoted to exploring the relationship between long non-coding RNAs (lncRNAs) and RA pathogenesis. Beyond their utility as RA biomarkers, lncRNAs are intricately involved in various pathological processes, encompassing inflammation, proliferation, migration, invasion, and apoptosis [12]. Notably, the LncRNA LERFS negatively regulates the invasion and proliferation of the RA synovium, offering potential therapeutic prospects for RA management [13]. Conversely, the under-expression of lncRNA DILC in RA, and the subsequent overexpression of DILC, has been suggested as a strategy to ameliorate RA by downregulating interleukin-6 (IL-6) and inhibiting fibroblast-like synoviocytes (FLSs) apoptosis [14]. Reduced FLS apoptosis is a key pathogenic mechanism in RA. A growing body of research has elucidated the involvement of lncRNAs in the capacity of serving as competing endogenous RNA (ceRNA) within the context of inflammatory processes and immune-related disorders [15, 16]. The ceRNA regulatory axis, composed of RNA transcripts, represents an emerging mode of gene expression regulation. Within this network, microRNAs (miRNAs) engage with messenger RNAs (mRNAs) via miRNA recognition elements (MREs), thereby effecting negative regulation of gene expression. Concurrently, lncRNAs, as endogenous entities, competitively interact with miRNAs through MREs to facilitate targeted mRNA expression [17]. Importantly, prior investigations have established a potential linkage between the ceRNA regulatory axis and the etiology of numerous diseases [18, 19]. An analysis of the ceRNA network has unveiled that NEAT1/MALAT1-hsa-miR-32-3p-SP1/FZD6 and NEAT1/MALAT1-hsa-miR-22-3p-PTEN/ESR1/ERBB3/CSF1R/CDK6 are likely pivotal contributors to the pathogenesis of osteoporosis [20].

**Fig. 1:**
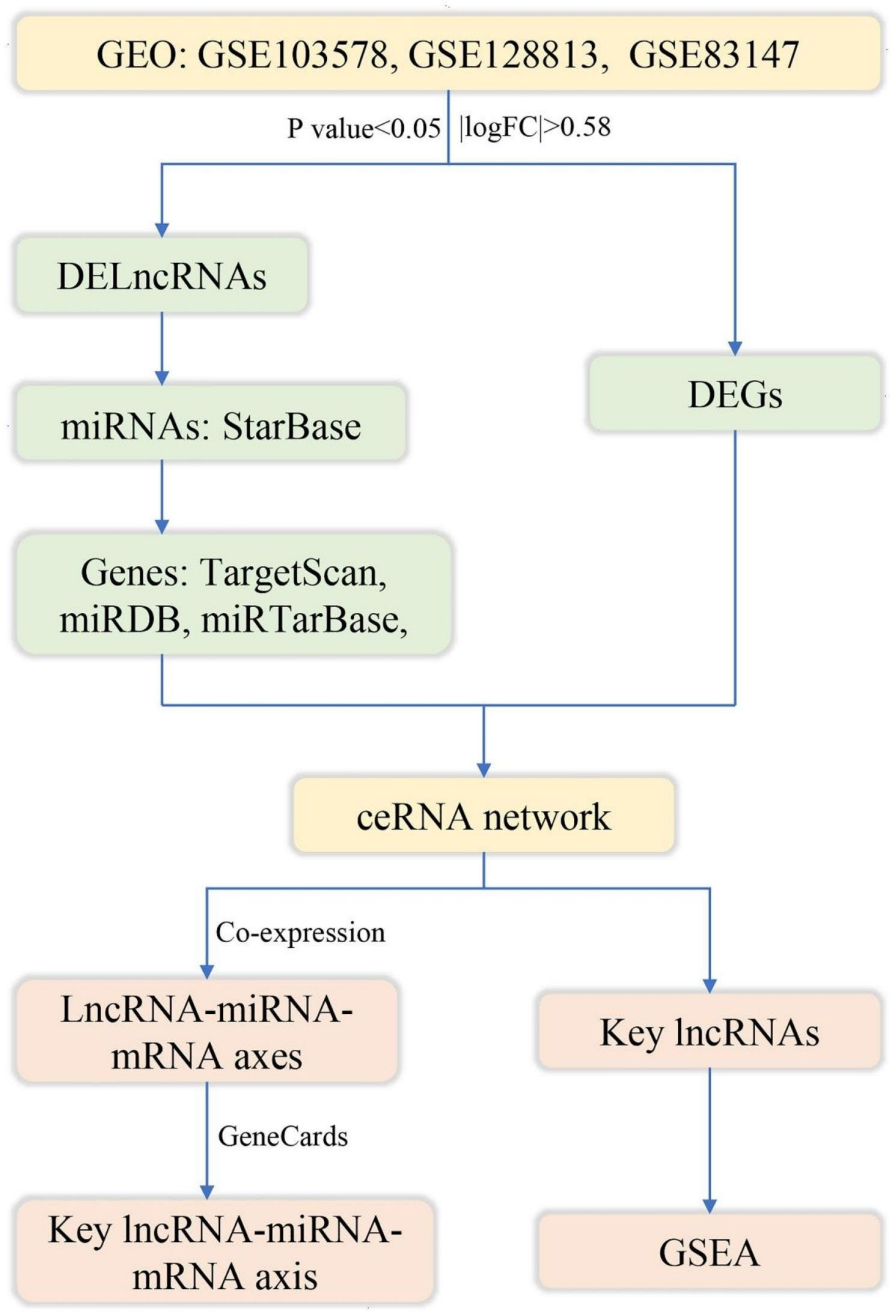
Technical strategy of the current study

In this study, we aimed to construct a ceRNA network of RA, screen the potential biomarkers and lncRNA-miRNA-mRNA axes in synovial lesions of RA, and conduct enrichment analysis of key lncRNAs. The aim of this study to offer novel research avenues and a foundational theoretical framework to advance our understanding of RA’s pathogenesis and the subsequent identification of therapeutic targets. A schematic representation of the technical approach employed in this study is depicted in Fig. 1.

## Materials and methods

### Microarray data

FLSs occupy a pivotal role in the pathogenesis of RA. This investigation sought to construct a ceRNA network involving RA-FLSs with the objective of elucidating the putative molecular contributors in the etiology of RA. To ensure the robustness of the established ceRNA network, it was imperative that both mRNAs and lncRNAs within the ceRNA network exhibit differential expression patterns in RA synovial tissue compared to normal synovial tissue. Consequently, the inclusion criteria for the microarray data employed in this study necessitated adherence to two stringent conditions. Firstly, the microarray data had to originate from both normal synovial tissue and RA synovial tissue samples. Secondly, the microarray dataset had to encompass both mRNAs and lncRNAs, facilitating subsequent differential expression analysis. Such stringent prerequisites considerably restricted the pool of eligible microarray data sources. Consequently, three microarray datasets of RA synovial tissue (GSE103578, GSE128813, and GSE83147) were downloaded from the Gene Expression Omnibus (GEO) database (http://www.ncbi.nlm.nih.gov/geo). GSE103578 comprised three RA synovial tissue samples and three normal synovial tissue samples, with its corresponding platform denoted as GPL15314. GSE128813 included three RA synovial tissue samples and three normal synovial tissue samples, with platform designation GPL21827. GSE83147 encompassed three RA synovial tissue samples and three normal synovial tissue samples, associated with platform GPL16956. The relatively small sample size employed in this study may potentially impact the outcomes, thus underscoring the necessity for further exploration and validation of the findings in future investigations.

### Data processing

Due to the existence of only one sequence within the platform files (GPL15314, GPL21827, GPL16956) associated with the microarray dataset, and the absence of gene symbols, it became imperative to undertake a process of re-annotation for these three platform files. The initial step involved the retrieval of the “gencode.v37.transcripts.fa” file, which contains comprehensive transcript information, from the Genecode database (https://www.gencodegenes.org/). Subsequently, the gene alignment software “seqmap-1.0.12-windows.exe” compatible with our computing environment was acquired. A Perl script was employed to convert the three platform files, namely GPL15314, GPL21827, and GPL16956, into their corresponding “fasta” file formats. Following the installation of “git for windows” on the computer system, gene alignment was executed via the command line interface, leveraging the “gencode.v37.transcripts.fa” reference file, the platform-specific “fasta” files, and the aforementioned comparison software, seqmap-1.0.12-windows.exe. Subsequent to the comparison process, the resultant output files were subjected to data extraction utilizing the “tidyr” and “dplyr” packages within the R environment (version 4.0.5). This procedure culminated in the successful retrieval of gene symbols within the platform files. Following the re-annotation of the platform files, a Perl script was employed to convert the gene identifiers within the three microarray datasets into corresponding gene symbols, facilitating subsequent differential analysis.

### Differential expression

The consolidation of three microarray datasets was achieved through the utilization of the Perl programming language. Subsequently, the “sva” and “limma” packages within the R (version 4.0.5) were employed for the purpose of removing inter-batch disparities. The “limma” package was also employed to identify differentially expressed lncRNAs (DELncRNAs) and differentially expressed genes (DEGs). The selection criteria applied for this differential expression analysis consisted of a significance threshold with a P value < 0.05 and |logFC|>1. In instances where the resultant set of DELncRNAs and DEGs, meeting these criteria, yielded an insufficient quantity, a more permissive threshold was considered, allowing for a P value < 0.05, and |logFC|>0.58 [21–24].

### Construction of ceRNA network

The StarBase database (http://starbase.sysu.edu.cn/starbase2/index.php) served as the primary resource for the prediction of miRNAs associated with DELncRNAs. Furthermore, for the prediction of target genes corresponding to these miRNAs, the miRDB database (http://mirdb.org/), miRTarBase database (http://mirtarbase.mbc.nctu.edu.tw/index.html), and TargetScan database (http://www.targetscan.org/vert_72/) were employed. To enhance the robustness and reliability of our predictions, we conducted an intersection analysis of the results obtained from the three aforementioned databases. Intersect the target genes identified for miRNAs with DEGs. Subsequently, the target miRNAs and lncRNAs were determined based on the common genes obtained from this intersection. Ultimately, the identified intersection genes, along with their associated targeted miRNAs and lncRNAs, were utilized in the construction of a ceRNA network using Cytoscape (version 3.8.0).

### Co-expression analysis of key lncRNAs and key genes

Co-expression analysis involves the establishment of gene relationships based on the congruence of gene expression data. Genes displaying concordant expression patterns are interconnected to establish a co-expression network. This co-expression network facilitates the identification of genes that exhibit coordinated expression patterns across a defined set of samples [25]. Subsequently, the lncRNAs and their corresponding target genes within the ceRNA network underwent co-expression analysis using the “limma” package in R. A |Pearson correlation coefficient|>0.4 and P value < 0.05 was employed to identify genes that are co-expressed with the key lncRNAs [26].

### Identification of immune-related genes

The GeneCards database (https://www.genecards.org/), serves as the preeminent hub for the comprehensive annotation of human genes. It boasts the capability to extract and amalgamate gene annotation data sourced from an expansive array of over 80 data repositories, including notable entities such as GTEx and BioGPS. Moreover, the database facilitates inquiries into the expression profiles of genes within diverse biological systems encompassing immune, nervous, muscular, internal, secretory, and reproductive systems gene annotation, which can automatically mine and integrate gene annotation information from [27, 28]. The GTEx project has made a pivotal contribution by furnishing RNA sequencing data derived from 54 non-pathological tissue sites, sourced from a cohort of nearly 1,000 individuals [29]. In parallel, BioGPS operates as a centralized gene repository, leveraging extant genetic and genomic resources to consolidate dispersed gene annotation information [30]. RA is an autoimmune disease, and the immune system plays a very important role in its pathogenesis. In our pursuit of discerning the potential associations between the co-expression genes of key lncRNAs and the immune system, we harnessed the wealth of information encapsulated within the GTEx and BioGPS databases. This was accomplished through the utilization of the online tool GeneCards, which facilitated the acquisition of expression scores within the immune system for genes, thus enabling the identification of genes exhibiting heightened correlations with RA [31].

### Identification of key lncRNA-miRNA-mRNA axis

Following the execution of a co-expression analysis involving key lncRNAs and their corresponding key genes, we obtained the co-expressed genes associated with these key lncRNAs. Subsequently, we integrated these key lncRNAs, their co-expressed genes, and the respective miRNAs into the lncRNA-miRNA-mRNA axis. To evaluate the significance of the co-expressed genes linked to key lncRNAs, we employed the GeneCards database to assign scores. Specifically, we focused on identifying the co-expressed genes of key lncRNAs that exhibited the highest expression levels within immune tissues. Within the lncRNA-miRNA-mRNA axes, we delineated the specific axis featuring co-expressed genes of key lncRNAs with the highest expression in immune tissues, thereby designating it as the key lncRNA-miRNA-mRNA axis relevant to RA.

### Gene set enrichment analysis (GSEA) of key lncRNAs

GSEA is a technology that is widely used for transcriptome data analysis. This approach leverages a predefined gene set database to systematically arrange gene lists derived from microarray, thereby identify significant and coordinated alterations in gene expression patterns. Subsequently, it discerns whether these expression profiles exhibit noteworthy enrichment pertaining to specific biological functions [32]. In our study, we utilized GSEA (version 4.0.3) to conduct Gene Ontology (GO) and Kyoto Encyclopedia of Genes and Genomes (KEGG) [33–35] enrichment analyses on key lncRNAs through the examination of their expression profiles. The primary objective was to explore the biological processes and signaling pathways that were significantly associated with the pathogenesis of RA. The selection criterion for significance was set at a P value < 0.05 [36–38]. The GO enrichment analysis provided annotations encompassing the biological process (BP), cellular component (CC), and molecular function (MF) associated with the identified key lncRNAs, while the KEGG analysis revealed the specific signal pathways in which these lncRNAs exhibited enrichment.

## Results

### Differential expression

**Fig. 2:**
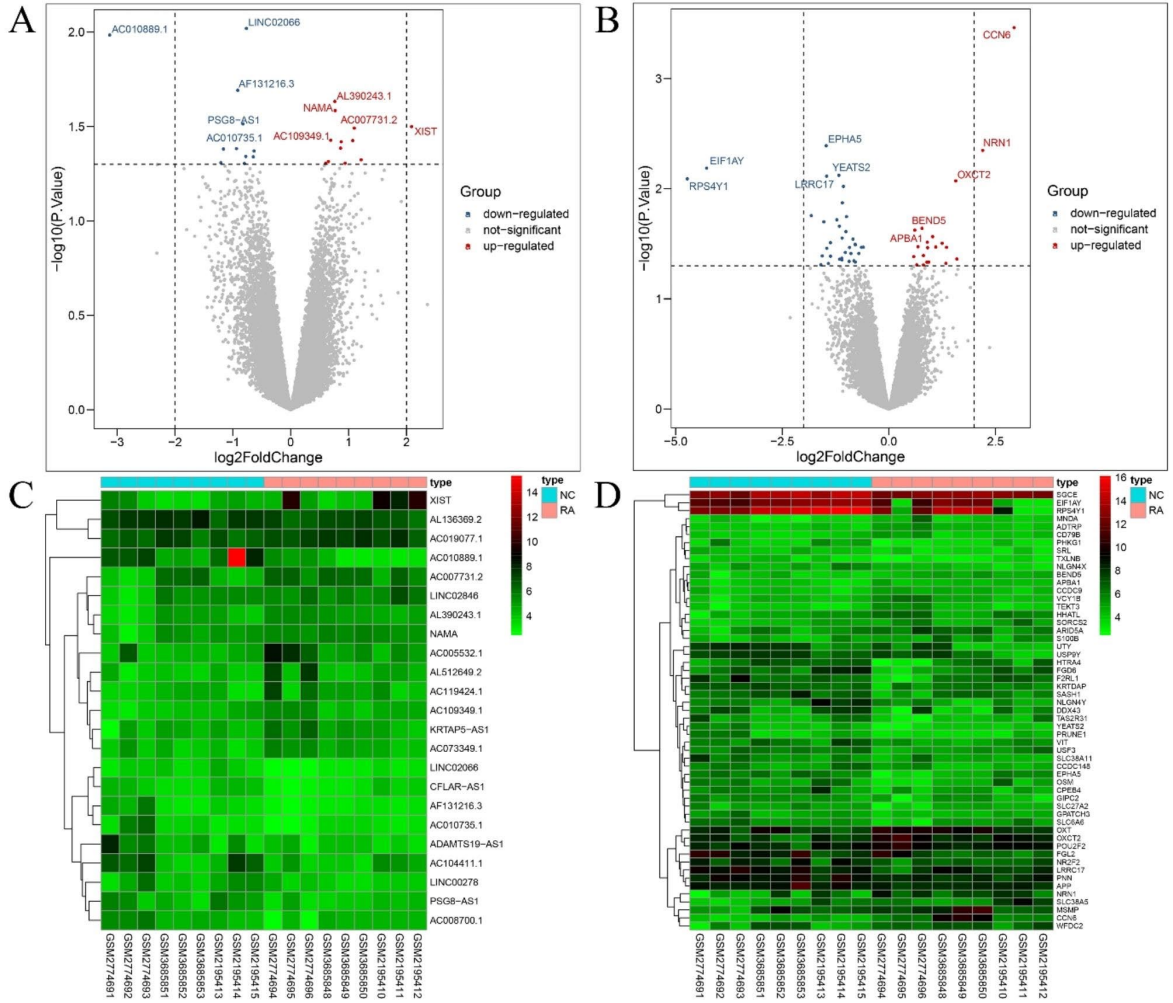
Differential analysis. **A**: The volcanomap of DELncRNAs, red are up-regulated DELncRNAs, blue is down-regulate, and grey are undifferentiated. **B**: The volcanomap of DEGs, red are up-regulated DEGs, blue is down-regulate, and grey is undifferentiated. **C**: Heatmap of DELncRNAs. Red is high expression, green is low expression. **D**: Heatmap of DEGs. Red is high expression, green is low expression

The differential analysis resulting in the identification of a total of 40 DELncRNAs and DEGs, comprising 7 DELncRNAs and 33 DEGs. Given the limited number of identified DELncRNAs and DEGs, there was a concern regarding the adequacy of DELncRNAs for downstream miRNA target prediction. Consequently, the analysis was expanded by relaxing the significance threshold to P value < 0.05, |logFC|>0.58, leading to the selection of 23 DELncRNAs and 55 DEGs. Among these, the 23 DELncRNAs included 12 upregulated and 11 downregulated DElncRNAs, while the 55 DEGs consisted of 20 upregulated and 35 downregulated DEGs. The visualization of the differential expression results was accomplished using the “ggpubr,“ “ggthemes,“ and “ggplot2” packages in R, resulting in the creation of volcano plots for DELncRNAs and DEGs (Fig. 2A and B). For clarity and aesthetics, only the top five upregulated and downregulated DELncRNAs and DEGs with the smallest P values were displayed in the volcano plots. Subsequently, heatmaps representing the expression patterns of DELncRNAs and DEGs were generated using the “pheatmap” package in R (Fig. 2C and D).

### Construction of ceRNA network

**Fig. 3:**
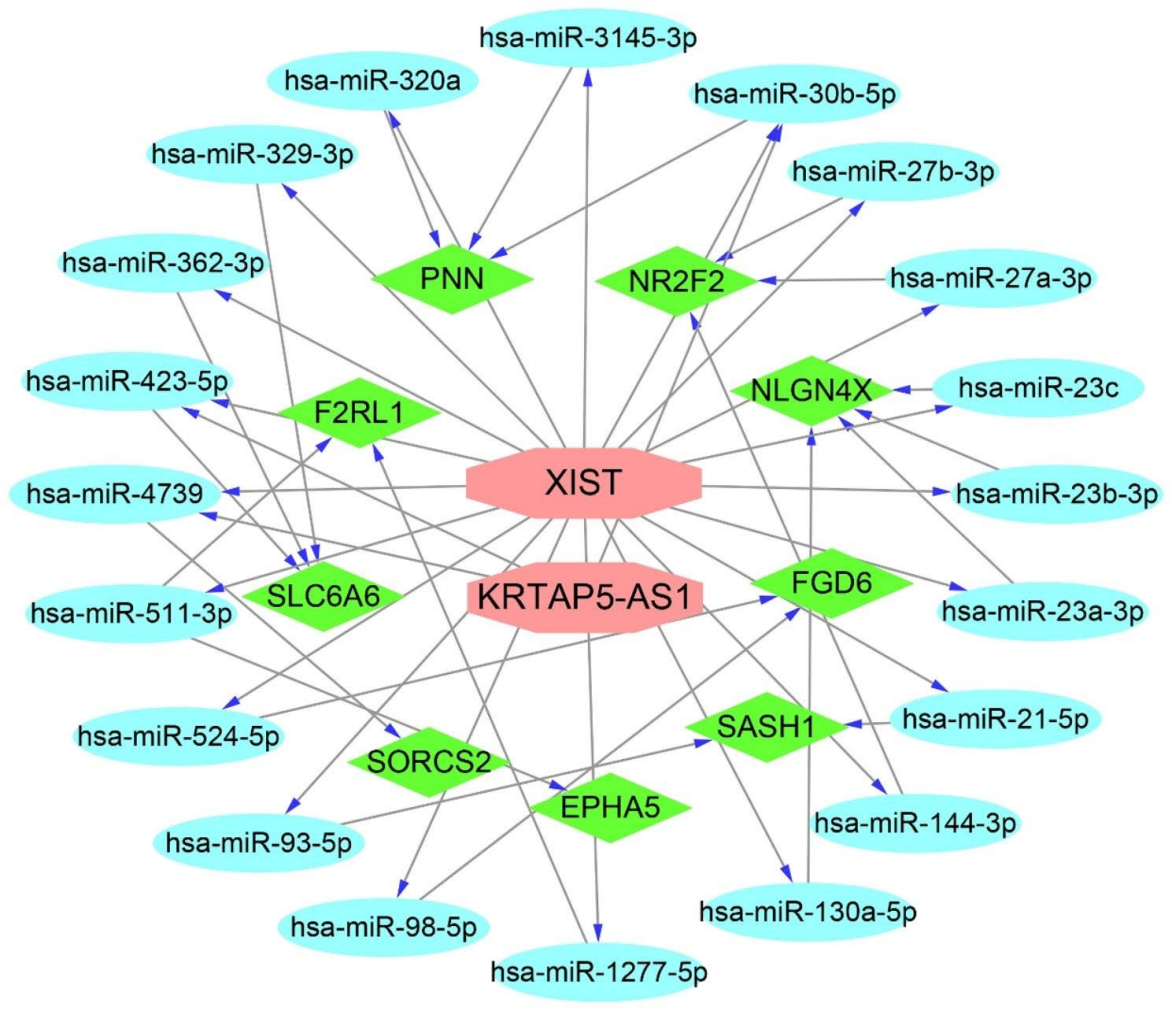
The network of ceRNA constructed by Cytoscape. Red is the key lncRNAs, blue is the key miRNAs, and green is the key gene

Five out of the 23 DELncRNAs were identified to potentially interact with 535 miRNAs as per the starBase database (Table 1). These 535 miRNAs were further analyzed to predict their respective target genes through a comprehensive query across three distinct databases, namely miRDB, miRTarBase, and TargetScan. The ensuing integration of data from these sources resulted in the identification of a substantial set comprising 14,778 target genes. A subsequent intersection of these 14,778 target genes 55 DEGs culminated in the identification of a select cohort of nine key genes. Utilizing these nine key genes, along with their associated miRNAs and DELncRNAs, we constructed a ceRNA network employing Cytoscape. The ceRNA network encompasses two key lncRNAs, 20 key miRNAs, and nine key genes (Fig. 3).

**Table 1:** The miRNAs corresponding to DELncRNAs.

DELncRNAs	Count of miRNAs	Database
XIST	446	starBase
AF131216.3	11	starBase
KRTAP5-AS1	59	starBase
AC010735.1	10	starBase
AC005532.1	9	starBase

DELncRNAs: differentially expressed LncRNAs

### Co-expression analysis of key lncRNAs and key genes

**Fig. 4:**
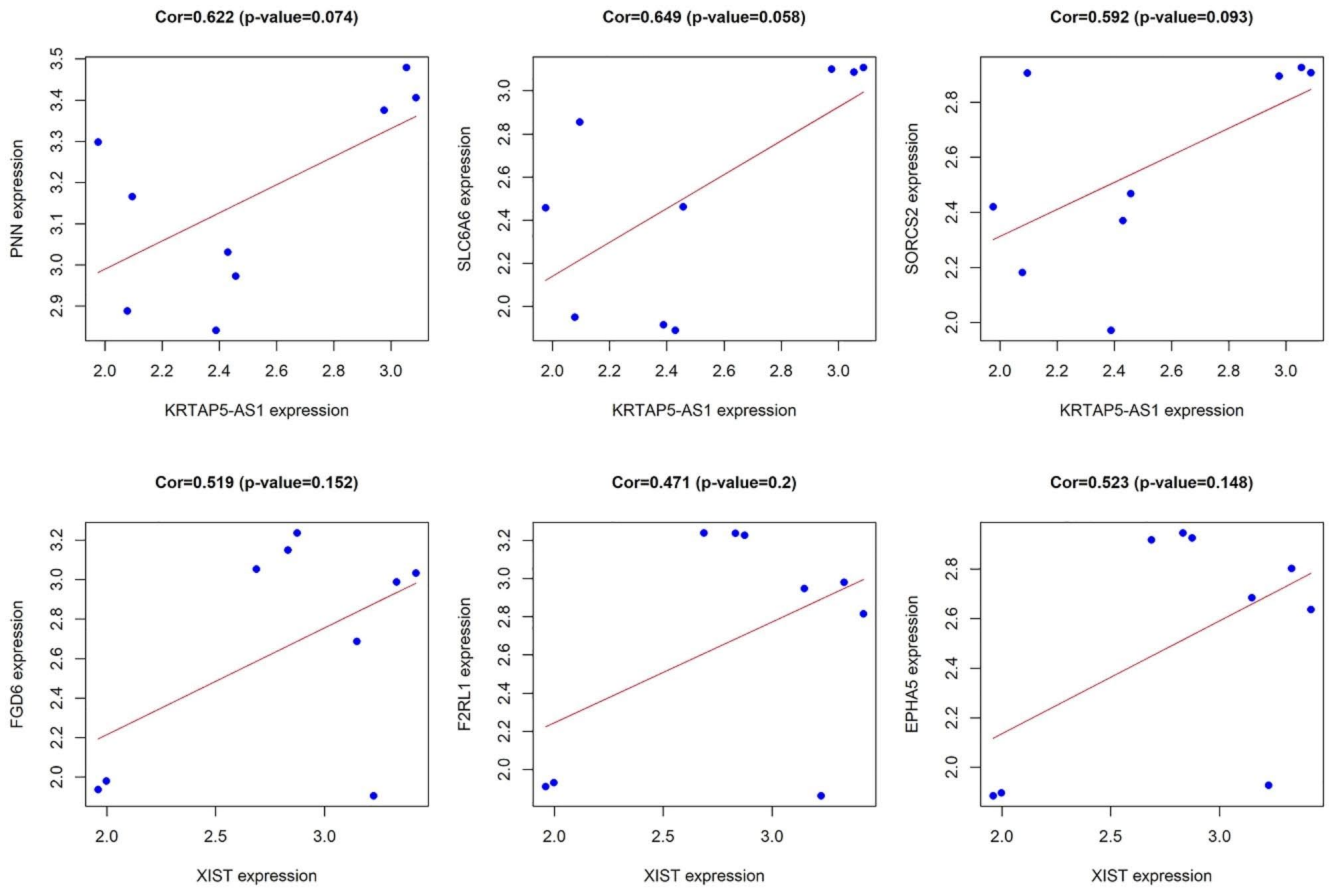
For co-expression analysis of key lncRNAs and key genes, the abscissa is lncRNAs and the ordinate is genes

From the ceRNA network analysis, it has been discerned that KRTAP5-AS1 exhibits regulatory interactions with three key genes, namely PNN, SLC6A6, and SORCS2. Furthermore, XIST has been identified as a regulator of nine key genes, including PNN, SLC6A6, SORCS2, NR2F2, FGD6, NLGN4X, F2RL1, SASH1, and EPHA5. The investigation performed the co-expression analysis between the identified key lncRNAs and their respective target genes. Initial screening based on the criteria of |Pearson correlation coefficient|>0.4 and P value < 0.05 yielded no significant results. Consequently, an expansion of the screening threshold to |Pearson correlation coefficient|>0.4 was executed [26], resulting in the identification of co-expression relationships. Specifically, KRTAP5-AS1 exhibited co-expression patterns with three key genes (PNN, SLC6A6, and SORCS2), while XIST demonstrated co-expression with three key genes (FGD6, F2RL1, and EPHA5) (Fig. 4). In our analysis, a total of eight lncRNA-miRNA-mRNA axes pertinent to RA were delineated, encompassing two key lncRNAs, seven key miRNAs, and six key genes (Table 2).

**Table 2:** LncRNA-miRNA-mRNA of RA.

Number	LncRNA-miRNA-mRNA
1	KRTAP5-AS1-hsa-miR-30b-5p-PNN
2	KRTAP5-AS1-hsa-miR-423-5p-SLC6A6
3	KRTAP5-AS1-hsa-miR-4739-SORCS2
4	XIST-hsa-miR-524-5p-FGD6
5	XIST-hsa-miR-98-5p-FGD6
6	XIST-hsa-miR-511-3p-F2RL1
7	XIST-hsa-miR-1277-5p-F2RL1
8	XIST-hsa-miR-511-3p-EPHA5

RA: Rheumatoid arthritis

### Identification of immune-related genes

**Fig. 5:**
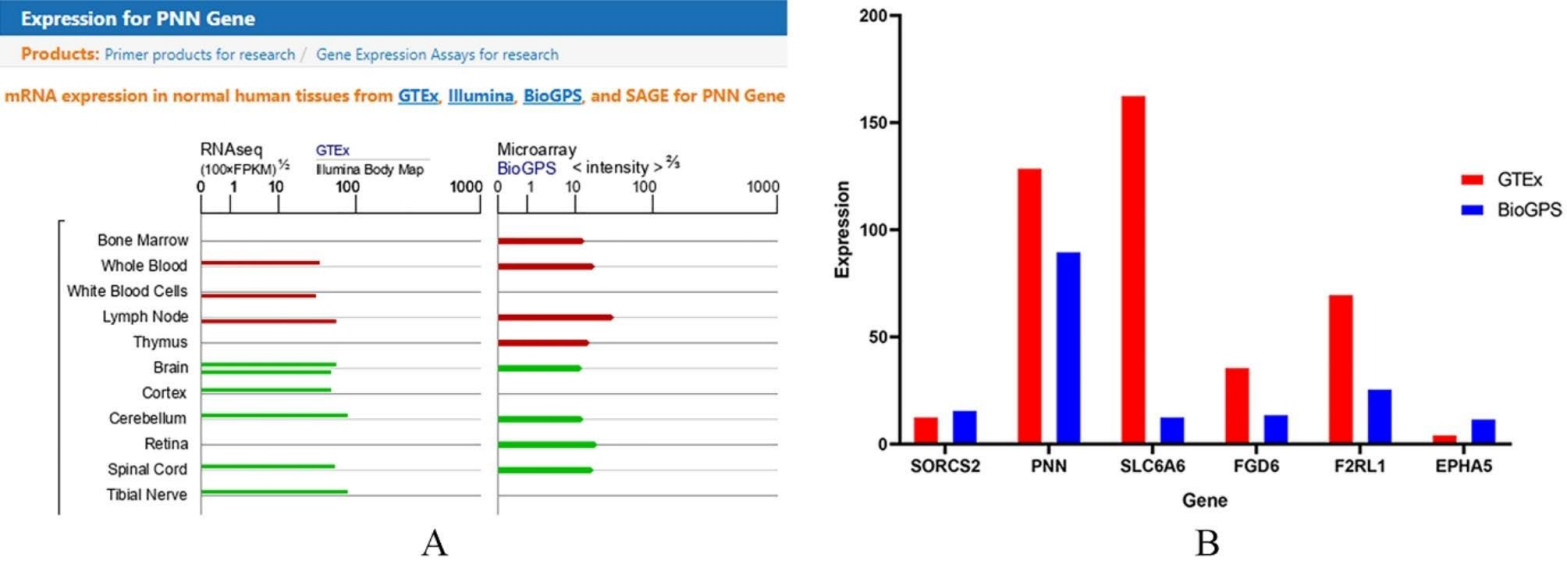
The expression levels of key genes in immune tissues. **A**: The key gene PNN search results in the GeneCards database, red is the expression level in immune tissues. **B**: The abscissa is the key gene, the ordinate is the expression level, the red is the expression level of the key gene in the GTEx database, and the blue is the expression level of the key gene in the BioGPS database

We leveraged data from the GTEx and BioGPS databases, accessible through GeneCards, to discern genes with enhanced relevance to the immune system [39]. We searched the PNN in the GeneCards database to determine its expression in each system, where dark red represents the immune system (Fig. 5A). Within the context of KRTAP5-AS1, an analysis of co-expressed genes (PNN, SLC6A6, and SORCS2) revealed PNN to exhibit the highest expression levels in immune tissues. Similarly, in the case of XIST, examination of co-expressed genes (FGD6, F2RL1, and EPHA5) identified F2RL1 as the gene with the highest expression within immune tissues (Fig. 5B). Based on these findings, we posit that PNN and F2RL1 are intimately associated with RA.

### Identification of key lncRNA-miRNA-mRNA axis

Within the context of RA, a total of eight lncRNA-miRNA-mRNA axes have been obtained. Among these, the axes KRTAP5-AS1-hsa-miR-30b-5p-PNN and XIST-hsa-miR-511-3p/hsa-miR-1277-5p-F2RL1 have been identified as key lncRNA-miRNA-mRNA axes that may play a significant role in the pathogenesis of RA.

### GSEA of key lncRNAs

**Fig. 6:**
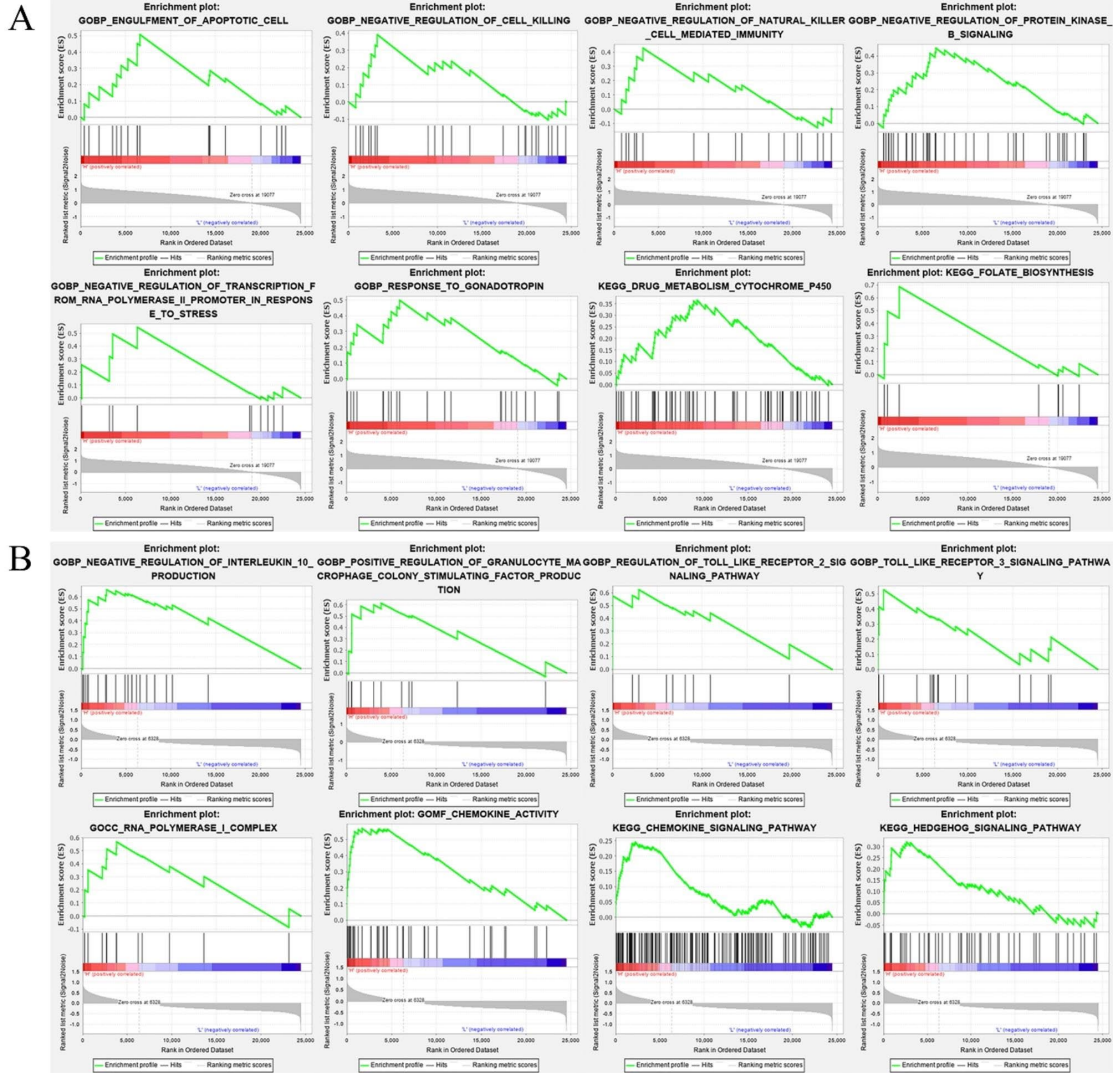
GSEA enrichment analysis of key lncRNAs, GO and KEGG enrichment analysis. The KEGG pathway database is copyrighted by Kanehisa laboratories and we have obtained the formal permission (KEGG_PERMISSION_231655) from them to publish this material commercially under an Open Access license. **A**: GSEA enrichment analysis of KRTAP5-AS1. **B**: GSEA enrichment analysis of XIST.

The results of the GSEA indicate that with respect to BP, KRTAP5-AS1 exhibits significant enrichment in various cellular functions, including the engulfment of apoptotic cells, negative regulation of cell killing, negative regulation of natural killer cell-mediated immunity, negative regulation of protein kinase B signaling, negative regulation of transcription from RNA polymerase II promoter in response to stress, and response to gonadotropin. In terms of signaling pathways, KRTAP5-AS1 predominantly demonstrates enrichment in the drug metabolism cytochrome p450 pathway and the folate biosynthesis pathway (Fig. 6A). With respect to BP, XIST displays significant enrichment in negative regulation of interleukin 10 production, positive regulation of granulocyte-macrophage colony-stimulating factor production, regulation of the Toll-like receptor 2 signaling pathway, and the Toll-like receptor 3 signaling pathway. Moreover, XIST is notably enriched in the RNA polymerase I complex within the CC category and exhibits chemokine activity in the MF category. With regard to signaling pathways, XIST exhibits significant enrichment in the chemokine signaling pathway and the Hedgehog signaling pathway (Fig. 6B).

## Discussion

Through the construction of the ceRNA network in the context of RA, we have delineated a comprehensive landscape encompassing eight distinct lncRNA-miRNA-mRNA axes that may intricately associated with RA. Within this network, we have identified two key lncRNAs, seven key miRNAs, and six noteworthy genes, all of which hold promise as prospective biomarkers for elucidating the etiological underpinnings of RA. Notably, our analysis has singled out two central lncRNA-miRNA-mRNA axes, specifically, KRTAP5-AS1-hsa-miR-30b-5p-PNN and XIST-hsa-miR-511-3p/hsa-miR-1277-5p-F2RL1, which represent putative RNA regulatory pathways instrumental in modulating the progression of RA. Subsequently, we conducted a GSEA to elucidate the functional implications of the aforementioned pivotal lncRNAs. Our analysis revealed that KRTAP5-AS1 is intricately linked to various biological processes and signaling pathways, including immune regulation, sex hormone regulation, protein kinase B signaling, apoptosis, and folic acid synthesis. Furthermore, XIST has been identified as a critical participant in signaling pathways such as the chemokine signaling pathway, Toll-like receptor signaling pathway, interleukin 10 signaling, and macrophage colony-stimulating factor signaling, all of which may pivotal in the intricate landscape of RA pathogenesis.

RA represents a clinical syndrome encompassing diverse disease subcategories, each characterized by distinct inflammatory cascades whose interplay culminates in persistent synovial inflammation, ultimately leading to cartilage degradation and joint deterioration [40]. A pivotal facet of this inflammatory cascade involves intricate interactions between T and B lymphocytes, FLSs, and macrophages, resulting in the excessive production and overexpression of tumor necrosis factor (TNF). This, in turn, induces the heightened secretion of various cytokines, notably IL-6, thereby perpetuating synovial inflammation and provoking joint destruction [41]. The articular swelling observed in RA stems from synovial inflammation driven by immune activation, characterized by the infiltration of white blood cells into synovial compartments that are typically sparsely populated [42]. The intricate regulatory network comprising cytokines and chemokines contributes significantly to the inflammatory milieu within the synovial space, with TNF, IL-6, and granulocyte-monocyte colony-stimulating factors playing pivotal roles within this regulatory framework [43]. Cytokines and chemokines not only activate endothelial cells but also prompt immune cell aggregation within the synovial cavity, thereby exacerbating the inflammatory response. Furthermore, cytokines stimulate chondrocytes to disrupt their catabolic processes, resulting in cartilage destruction, while the cartilage matrix is degraded by enzymes such as matrix metalloproteinase (MMP) [42]. The pathogenesis of RA involves the active participation of various cellular components, including synovial cells, fibroblasts, lymphocytes, and chondrocytes, collectively contributing to the progression of the disease. Notably, RA manifests as a systemic ailment affecting multiple tissues, and it is important to acknowledge that findings derived from our investigation on synovial tissue may not comprehensively represent the entirety of the RA disease trajectory.

KRTAP5-AS1, a genomic locus situated on chromosome 11 spanning 2554 nucleotides [44], has emerged as a promising biomolecular entity in the context of Hepatitis B virus-associated hepatocellular carcinoma (HBV-HCC). This RNA molecule appears to exert a dualistic influence on the etiology and progression of HBV-HCC, potentially functioning in either a protective or risk-enhancing capacity, contingent upon its co-expression with MC1R [45]. Furthermore, KRTAP5-AS1 has garnered attention as a diagnostic marker in papillary thyroid carcinoma (PTC), where its expression levels are intricately linked with the prognosis of this malignancy [46]. In the sphere of gastric cancer (GC), KRTAP5-AS1 assumes a multifaceted role by acting as a ceRNA, thereby modulating the functionality of the Claudin-4 network (CLDN4). It also serves as an oncogenic element by virtue of its interaction with miR-596 and miR-36203p in in vitro settings [44]. The PNN gene, encoding the pinin protein, initially recognized for its pivotal role in epithelial cell adhesion, is intricately involved in cellular adhesion processes and mRNA splicing mechanisms mediated through the spliceosome [47]. The abatement of PNN expression exerts a regulatory influence on splicing factor levels and governs alternative pre-mRNA splicing events in vivo [48]. Notably, heightened PNN expression levels have been documented in gastrointestinal neoplasms, rendering it a viable independent prognostic indicator and immunotherapeutic response predictor in colon adenocarcinoma (COAD) [47]. Furthermore, the PNN gene features prominently in the spliceosome-associated pathways of prostate cancer (PCa) and has been identified as a potential diagnostic marker for PCa [49]. Beyond its role in cancer, PNN plays a critical role in preserving astrocytic vitality, sustaining anti-apoptotic capability, and bolstering mitochondrial bioenergetic functions, thus averting astrocytic cell demise following acute ischemic stroke [50]. Additionally, PNN exhibits a dichotomous function in cancer biology by promoting cell proliferation and impeding apoptosis in liver cancer, while concurrently facilitating carcinogenic processes in renal cell carcinoma by curtailing apoptosis and enhancing cell migration and invasion [51, 52]. Furthermore, the interaction between hsa-circ-0032463 and PNN assumes significance in the realm of osteosarcoma (OS), with hsa-circ-0032463 acting as a potent promoter of tumorigenesis via regulation of the miR-330-3p/PNN axis. Both hsa-circ-0032463 and PNN manifest elevated expression levels in OS tissues, thereby potentiating OS cell proliferation, invasion, adhesion, and attenuating apoptosis [53]. Evidently, PNN emerges as a pivotal player in the pathogenesis of various diseases by virtue of its capacity to mitigate apoptosis. Intriguingly, apoptosis is intricately entwined with RA, involving aberrant apoptotic processes across diverse cellular components, including synovial cells, fibroblasts, lymphocytes, and chondrocytes, thereby driving synovial hyperplasia and hypertrophy and contributing to the pathogenesis of RA. While the specific correlation between KRTAP5-AS1, PNN, and RA remains uncharted in extant literature, prior investigations allude to the plausible hypothesis that KRTAP5-AS1 may exert a regulatory influence on the initiation and progression of RA via co-expression with PNN. PNN may induce synovial hyperplasia and hypertrophy by reducing synovial cell apoptosis, thus promoting the occurrence and development of RA. Additionally, KRTAP5-AS1 may function as a ceRNA, potentially modulating the regulatory KRTAP5-AS1-hsa-miR-30b-5p-PNN axis in the context of RA. Substantiation of these conjectures necessitates further exhaustive study.

XIST represents a cell-specific, nuclear-encoded lncRNA, which assumes a pivotal role in X chromosome inactivation. Notably, mutations affecting the XIST promoter have been implicated in familial instances of skewed X chromosome inactivation. Furthermore, XIST has demonstrated the capability to modulate acute inflammation [54], and this is particularly pertinent in the context of RA, a prototypical inflammatory ailment. Research has ascertained heightened XIST expression within RA cartilage tissue, alongside diminished levels of let-7c-5p in the same context. Pertinently, interventions such as XIST silencing and let-7c-5p augmentation have been shown to ameliorate inflammation by reducing the levels of pro-inflammatory cytokines, including TNF-α, IL-2, and IL-6, thereby concomitantly enhancing the levels of alkaline phosphatase (ALP), osteocalcin, transforming growth factor-β1 (TGF-β1), and insulin-like growth factor-1 (IGF-1), with mitigating cartilage tissue damage [55]. Furthermore, XIST appears to exert a regulatory influence upon osteoblast proliferation and differentiation by modulating the STAT3 pathway, thus engendering an association with the pathogenesis of RA [55]. Additionally, prior investigations have spotlighted XIST as a potential biomarker within the context of RA synovial lesions [39]. The F2RL1 gene encodes a member of the G-protein coupled receptor 1 family of proteins. Activation of this cell surface receptor is contingent upon proteolytic cleavage of its extracellular amino terminus, leading to the exposure of a tethered ligand that binds to an extracellular loop domain. Activation of F2RL1 has been demonstrated to engender vascular smooth muscle relaxation, vasodilation, increased blood flow, and reduced blood pressure, underscoring its pivotal role in inflammatory, innate, and adaptive immune responses. Moreover, F2RL1 has been identified as a reliable immune-related biomarker with prognostic significance in pancreatic cancer (PC) and colorectal cancer (CRC) [56, 57]. F2RL1 is intrinsically associated with signaling cascades potentiated by the interaction of low-density lipoproteins (LDL) with macrophages and is further implicated in the promotion of diet-induced obesity and fat inflammation [58, 59]. Notably, F2RL1 assumes an immunomodulatory role in models of inflammatory arthritis, thus promulgating the concept that F2RL1 antagonists may hold therapeutic promise in the management of inflammatory arthritis [60]. Serum analyses in patients afflicted with RA have revealed elevated levels of the activation fragment of F2RL1, which, crucially, exhibit a decrement in response to anti-IL6 receptor (IL6R) treatment. This highlights the potential utility of F2RL1 as a biomarker for monitoring the course of RA and pharmacodynamic responses to treatment [61]. F2RL1 activation has been shown to augment the growth and invasiveness of RA-FLSs, concomitantly increasing TNF production and exacerbating the pathological progression of RA [62]. Moreover, F2RL1 has been identified as a target gene of TGF-β [63], a molecule intimately associated with the pathogenesis of RA. Specifically, TGF-β1 has been implicated in promoting the migration and invasion of FLSs in the context of RA through the TGF-β1/Smad signaling axis [64]. Notably, deficits within the TGF-β1 signaling pathway are associated with diminished expression of CD39 in regulatory T cells and subsequent resistance to methotrexate therapy in the context of RA [65]. In summary, XIST emerges as a multifaceted player within the realm of RA pathobiology, serving as a potential biomarker for RA synovial lesions and exhibiting pronounced expression within RA cartilage tissue. Down-regulation of XIST stands to abate the inflammatory response in RA by mitigating levels of TNF-α, IL-2, and IL-6, thereby curbing cartilage tissue damage and retarding the progression of this debilitating condition. In parallel, F2RL1 assumes a role in promoting the aggressiveness of RA-FLSs, with its down-regulation offering the potential to ameliorate synovial inflammation in RA. Furthermore, XIST appears to be intricately involved in the pathogenesis of RA as a ceRNA, modulating the XIST-hsa-miR-511-3p/hsa-miR-1277-5p-F2RL1 axis.

This study has several noteworthy limitations. Firstly, the study’s sample size was relatively modest, and the employed sampling methodology did not fully account for confounding variables such as age, gender, and underlying medical conditions. Secondly, the functional roles of the putative biomarkers and pivotal lncRNA-miRNA-mRNA axes identified in this investigation with regards to the pathogenesis of RA have not been experimentally validated. It is imperative to undertake further molecular experiments in subsequent research endeavors to elucidate their prospective contributions to the etiology, diagnosis, and therapeutic management of RA. Thirdly, RA manifests as a systemic ailment impacting multiple tissues. Given the intricate and multifaceted nature of RA, it is prudent to acknowledge that outcomes derived from an exclusive focus on synovial tissue may not comprehensively encapsulate the entirety of the disease pathophysiology.

## Conclusions

Our research not only explores the prospective ceRNA regulatory network potentially associated with lncRNA in RA, but also posits possible biomarkers, delineates underlying biological processes, and delineates pathways implicated in the etiological framework of RA. This contribution augments the corpus of knowledge in the field, underscoring its significance for advancing the understanding of RA pathogenesis.

### Electronic supplementary material

Below is the link to the electronic supplementary material.

Supplementary Material 1

## Data Availability

The data supporting the results of the study are available from the GEO database (https://www.ncbi.nlm.nih.gov/geo/).
