# Supplementary material for: Identification of the potential regulatory interactions in rheumatoid arthritis through a comprehensive analysis of lncRNA-related ceRNA networks
Source: BMC Musculoskelet Disord. 2023 Oct 9;24:799. doi: 10.1186/s12891-023-06936-3 (PMC10561475; doi:10.1186/s12891-023-06936-3)
Supplement: Supplementary file 1 — Supplementary Material 1 [file 12891_2023_6936_MOESM1_ESM.docx]

**Difference analysis**

#source("http://bioconductor.org/biocLite.R")

#biocLite("limma")

logFoldChange=1

adjustP=0.05

library(limma)

setwd("")

rt=read.table("normalize.txt",sep="\t",header=T,check.names=F)

rt=as.matrix(rt)

rownames(rt)=rt[,1]

exp=rt[,2:ncol(rt)]

dimnames=list(rownames(exp),colnames(exp))

rt=matrix(as.numeric(as.matrix(exp)),nrow=nrow(exp),dimnames=dimnames)

#differential

modType=c(rep("normal",3),rep("tumor",3),rep("normal",3),rep("tumor",3),rep("normal",3),rep("tumor",3))

design <- model.matrix(~0+factor(modType))

colnames(design) <- c("con","treat")

fit <- lmFit(rt,design)

cont.matrix<-makeContrasts(treat-con,levels=design)

fit2 <- contrasts.fit(fit, cont.matrix)

fit2 <- eBayes(fit2)

allDiff=topTable(fit2,adjust='fdr',number=200000)

write.table(allDiff,file="limmaTab.xls",sep="\t",quote=F,row.names=F)

#write table

diffSig <- allDiff[with(allDiff, (abs(logFC)>logFoldChange & P.Value < adjustP )), ]

write.table(diffSig,file="diff.xls",sep="\t",quote=F,row.names=F)

diffUp <- allDiff[with(allDiff, (logFC>logFoldChange & P.Value < adjustP )), ]

write.table(diffUp,file="up.xls",sep="\t",quote=F,row.names=F)

diffDown <- allDiff[with(allDiff, (logFC<(-logFoldChange) & P.Value < adjustP )), ]

write.table(diffDown,file="down.xls",sep="\t",quote=F,row.names=F)

#write expression level of diff gene

hmExp=rt[as.vector(diffSig[,1]),]

diffExp=rbind(id=colnames(hmExp),hmExp)

write.table(diffExp,file="diffExp.txt",sep="\t",quote=F,col.names=F)

#volcano

pdf(file="vol.pdf")

xMax=max(-log10(allDiff$adj.P.Val))

yMax=max(abs(allDiff$logFC))

plot(-log10(allDiff$adj.P.Val), allDiff$logFC, xlab="-log10(adj.P.Val)",ylab="logFC",

main="Volcano", xlim=c(0,xMax),ylim=c(-yMax,yMax),yaxs="i",pch=20, cex=0.8)

diffSub=subset(allDiff, adj.P.Val<adjustP & logFC>logFoldChange)

points(-log10(diffSub$adj.P.Val), diffSub$logFC, pch=20, col="red",cex=0.8)

diffSub=subset(allDiff, adj.P.Val<adjustP & logFC<(-logFoldChange))

points(-log10(diffSub$adj.P.Val), diffSub$logFC, pch=20, col="green",cex=0.8)

abline(h=0,lty=2,lwd=3)

dev.off()

**Co-expression analysis**

## try http:// if https:// URLs are not supported

#source("https://bioconductor.org/biocLite.R")

#biocLite("limma")

setwd("")

inputFile="biotype.txt"

gene="LINC00526|lincRNA"

picDir="picture"

dir.create(picDir)

library(limma)

rt=read.table(inputFile,sep="\t",header=T,check.names=F)

rt=as.matrix(rt)

rownames(rt)=rt[,1]

exp=rt[,2:ncol(rt)]

dimnames=list(rownames(exp),colnames(exp))

data=matrix(as.numeric(as.matrix(exp)),nrow=nrow(exp),dimnames=dimnames)

data=avereps(data)

group=sapply(strsplit(colnames(rt),"\\-"),"[",4)

group=sapply(strsplit(group,""),"[",1)

group=gsub("2","1",group)

rt=rt[,group==0]

rt=data[rowMeans(data)>0,]

x=log2(as.numeric(rt[gene,])+1)

gene1=unlist(strsplit(gene,"\\|",))[1]

outputFile=paste(gene1,".cor.xls",sep="")

outTab=data.frame()

for(j in rownames(rt)){

y=log2(as.numeric(rt[j,])+1)

gene2=unlist(strsplit(j,"\\|",))[1]

gene2Type=unlist(strsplit(j,"\\|",))[2]

if(gene2Type=="protein_coding"){

corT=cor.test(x,y)

gene1Name=unlist(strsplit(gene1,"\\|",))[1]

gene2Name=unlist(strsplit(gene2,"\\|",))[1]

z=lm(y~x)

cor=corT$estimate

cor=round(cor,3)

pvalue=corT$p.value

if(pvalue<0.001){

pval=signif(pvalue,4)

pval=format(pval, scientific = TRUE)

}else{

pval=round(pvalue,3)}

if((abs(cor)>0.4) & (pvalue<0.001)){

tiffFile=paste(gene1Name,"_",gene2Name,".cor.tiff",sep="")

outTiff=paste(picDir,tiffFile,sep="\\")

tiff(file=outTiff,width =12,height = 12,units ="cm",compression="lzw",bg="white",res=300)

plot(x,y, type="p",pch=16,col="blue",main=paste("Cor=",cor," (p-value=",pval,")",sep=""),

cex=1， cex.lab=1, cex.main=1,cex.axis=1,

xlab=paste(gene1Name,"expression"),

ylab=paste(gene2Name,"expression") )

lines(x,fitted(z),col=2)

dev.off()

}

outTab=rbind(outTab,cbind(gene1,gene2,gene2Type,cor,pvalue))

}

}

write.table(file=outputFile,outTab,sep="\t",quote=F,row.names=F)
